# Cells/colony motion of oral keratinocytes determined by non-invasive and quantitative measurement using optical flow predicts epithelial regenerative capacity

**DOI:** 10.1038/s41598-021-89073-y

**Published:** 2021-05-17

**Authors:** Emi Hoshikawa, Taisuke Sato, Kenta Haga, Ayako Suzuki, Ryota Kobayashi, Koichi Tabeta, Kenji Izumi

**Affiliations:** 1 Division of Biomimetics, Faculty of Dentistry and Graduate School of Medical and Dental Sciences, Niigata University, Niigata, Japan; 2 Division of Periodontology, Faculty of Dentistry and Graduate School of Medical and Dental Sciences, Niigata University, Niigata, Japan; 3 Center for Transdisciplinary Research, Institute for Research Promotion, Niigata University, Niigata, Japan; 4 Division of Pediatric Dentistry, Faculty of Dentistry and Graduate School of Medical and Dental Sciences, Niigata University, Niigata, Japan

**Keywords:** Biological techniques, Cell biology

## Abstract

Cells/colony motion determined by non-invasive, quantitative measurements using the optical flow (OF) algorithm can indicate the oral keratinocyte proliferative capacity in early-phase primary cultures. This study aimed to determine a threshold for the cells/colony motion index to detect substandard cell populations in a subsequent subculture before manufacturing a tissue-engineered oral mucosa graft and to investigate the correlation with the epithelial regenerative capacity. The distinctive proliferating pattern of first-passage [passage 1 (p1)] cells reveals the motion of p1 cells/colonies, which can be measured in a non-invasive, quantitative manner using OF with fewer full-screen imaging analyses and cell segmentations. Our results demonstrate that the motion index lower than 40 μm/h reflects cellular damages by experimental metabolic challenges although this value shall only apply in case of our culture system. Nonetheless, the motion index can be used as the threshold to determine the quality of cultured cells while it may be affected by any different culture conditions. Because the p1 cells/colony motion index is correlated with epithelial regenerative capacity, it is a reliable index for quality control of oral keratinocytes.

## Introduction

The ability to generate functional equivalents as replacements for damaged or diseased human tissues is rapidly advancing in the fields of tissue engineering and regenerative medicine, which has harnessed the unlimited potential of mono-potent and multi-potent stem cells. By using transplantable tissues/organs manufactured from either autologous or allogeneic stem cells, regenerative technologies have contributed to cure various illnesses and injuries^1,2^. When culturing cells for relatively long periods, both the measurement and the evaluation of cell growth and differentiation potential are crucial to maintaining stable and reliable quality-assured cells for cell-based therapies. To meet the increased demand, quality control (QC) of engineered tissues and organs for clinical applications has become critical to confirm safety and effectiveness for the outcomes of post-grafting^3^. However, prior to grafting, conventional cellular evaluation techniques based on molecular biology remain invasive, costly and time consuming. Furthermore, these techniques are incompatible with clinical applications in regenerative medicine because of the inevitable damage or loss of observed cells^4^. More importantly, intact cells must be used in clinical settings. Thus, a non-invasive, low-cost and rapid cell evaluation method is required to expand the possibilities of regenerative cell-based therapy^5^. To address such challenges, several types of novel image-based analyses that integrate computational data processing can be applied to a variety of imaging systems and evaluated for efficacy^6^. These procedures allow investigators to monitor and quantitate specific states of cells during culturing in a non-invasive and high-throughput manner^7–9^.

Autologous oral mucosa keratinocytes have been employed in clinical arenas, not only in regenerative dentistry but also in extra-oral regenerative medicine^10–15^. Thus, manufacturing protocols for tissue-engineered constructs using oral keratinocytes are required to confront regulatory challenges pertaining to safety, efficacy, quality etc. The manufacturing protocol comprises two major steps. The initial step establishes a two-dimensional (2D) monolayer cell culture by isolating and culturing cells from living tissue. Next, using a three-dimensional (3D) framework, biomimicking multi-cellular engineered grafts are manufactured as final products for human use. Given that the criteria for the release of final products should be determined immediately before transplantation, real-time and non-invasive examinations of the 3D constructs are crucial. Recently, an advanced imaging system was introduced to evaluate complex 3D cell-based constructs^16,17^. Chen et al. previously reported a non-invasive optical assessment for oral mucosa tissue-engineered constructs using optical molecular microscopy by which metabolic activities were characterised^18,19^. However, because QC needs to be implemented throughout the entire period of cell culture, the state of oral keratinocytes, including proliferation and colony formation before seeding cells onto the scaffold, is critical for manufacturing the quality of cellular-based products. Hence, optimal non-invasive cell measurements are essential for oral keratinocyte regenerative medicine.

Optical flow (OF) is a popular image-based analysis that demonstrates the distribution of the apparent motion of brightness patterns between two consecutive frames, which is caused by the movement of objects^20^. Since the OF algorithm is associated with the motility of objects, previous studies have been conducted to assess the ‘motility’ related to specific cell functions, including research on cellular biology^21–24^ and cardiology^25,26^. Regarding cardiologic regenerative medicine, OF-based analyses have served as a non-invasive evaluation tool for the QC of cardiomyocytes to monitor contractile function without compromising the condition of the cells^27^. OF was also introduced to measure the collective motion speed of keratinocytes to identify skin stem cell colonies that were grown in the culture system with feeder layer cells^28^. Similar to that study, we reported that both the OF and normalised cross-correlation (NCC) can be leveraged to evaluate the proliferative capacity of primary oral keratinocytes within a relatively small-sized colony base during an early phase of cell culture^29^.

To obtain a sufficient number of healthy cells to manufacture 3D cell-based constructs, serial cell cultivation (subculture) from the primary culture, referred to as p0 cells, is indispensable because p0 cells subcultured to the first (p1), second (passage 2) and third (passage 3) passage cultures yield a large number of cells with exponential cell growth^10,11^. It is well-known that the p1 cells/colonies are likely to grow discretely and localise in small, loosely connected colonies, mainly because of a higher plating rate when seeded as p1 cells^30,31^, which results in the rapid proliferation of p1 cells, whereas p0 cells in the feeder layer and serum-free culture take a longer time to reach a higher confluency^32–36^. Although our previous study demonstrated the applicability and feasibility of the OF algorithm to non-invasively measure p0 oral keratinocyte cells/colony motion, the spatio-temporal growth kinetics of p1 oral keratinocyte cells/colonies in a 2D monolayer culture is much different from that of p0 cells. Such an expansive analysis requires an entire microscopic image to target and observe a larger number of cells/colonies, instead of a single-colony base. Since our previous image analyses were not efficient to measure the full extent of the cell area, an improved protocol was needed to implement the time-lapse phase-contrast microphotography to p1 cells^29^.

This study aimed to improve and enhance our previous protocol for the OF algorithm and to add the capability of determining the threshold of the cells/colony motion speed to differentiate substandard p1 oral keratinocyte populations before manufacturing a tissue-engineered oral mucosa tissue construct. By determining the specific spatio-temporal proliferating pattern of p1 cells, such as rapid growth and narrow space between neighbouring colonies, we could set correlative estimates that reduced the time of microphotography from 24 to 4 h, thereby resulting in fewer image analyses (31 frames compared to 96). Next, the image segmentation under full-screen analysis was used to identify the areas of cells/colonies under the microscopic images before applying the OF algorithm. Hence, 50% confluency was set to start the time-lapse microphotography and assess the overall cells/colony growth and motion speed (MS) of each frame by applying the OF algorithm. We then examined the applicability of the mean motion speed (MMS) to monitor the proliferative capacity of p1 oral keratinocytes by calculating the population doubling times (PDTs) and determining the threshold of MMS that is suitable for transfer to a 3D culture condition non-invasively and quantitatively as a QC tool. Finally, we tested whether the MMS of p1 cells can be used as an index to predict the epithelial regenerative capacity and evaluate histologic features of the tissue-engineered constructs after the p1 oral keratinocytes had received experimental metabolic challenges during a 2D culture condition, thereby demonstrating the threshold of the motion index of the p1 cells to determine whether their proliferation was adequate for manufacturing constructs.

## Results

### Validation of the time-lapse microphotography protocol for p1 cells/colony using the OF algorithm with image segmentation

Since the spatio-temporal growth (proliferating) kinetics of p1 oral keratinocyte cells/colonies differs from that of p0 cells, mainly due to the plating efficiency, a different protocol of non-invasive measurements was applied in this study. The protocol included time-setting [4 h at an interval of 8 min (31 frames)] for time-lapse microphotography and full-screen analysis before applying the OF algorithm.

**Figure 1:**
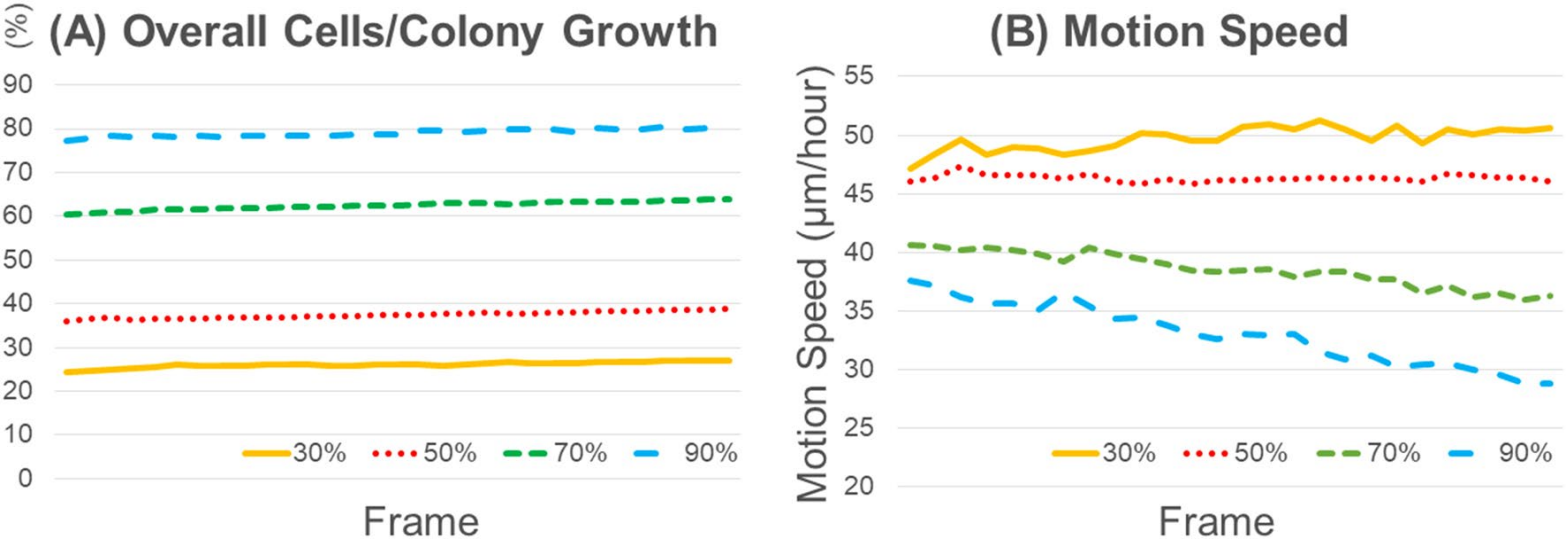
Characteristics of p1 oral keratinocyte cells/colonies growth in space and time. Representative overall cells/colony growth (**A**) and motion speed (MS) (**B**) for 4 h (31 frames) depending on four different initial cell confluencies under manual observation (30%, 50%, 70% and 90%). This data was used to determine what cell growth conditions were appropriate to start the time-lapse microphotography in this study. (**A**) Representative changes in overall cells/colony growth of p1 oral keratinocytes. The overall cells/colony growth was almost unchanged over time regardless of the initial cell confluency. (**B**) Representative changes of MS of p1 oral keratinocytes over time. MSs showed different patterns depending on the four different initial cell confluencies (30%, 50%, 70% and 90%).

To determine what starting condition was appropriate to implement non-invasive measurement for the p1 cells in this protocol, the overall cells/colony growth was investigated. It increased very slowly during 4 h in a similar fashion when time-lapse microphotography started at 30%, 50%, 70% and 90% confluency under manual observation, which indicated that the time-setting of 4 h is appropriate (Fig. 1A). On the other hand, the MS continued to decline over the period of 4 h when started at 70% and 90% confluency, while it remained unchanged at 50% confluency and showed a slight increase at 30% confluency during the time-lapse imaging (Fig. 1B). Since the MMS is a parameter of the mean of the total frames (31), the increase and decrease of the MS during microphotography is not appropriate as the representative data of the sample. Thus, as a new protocol, the starting conditions for time-lapse microphotography when the cells reached approximately 50% confluency was set and applied to further measurements used in this study.

Using the same OF algorithm as our previous report and different image segmentation, the motion behaviour of the individual p1 cells/colonies was successfully quantified, which indicated that MS can be applicable and feasible for non-invasive and quantitative measurements of the cells/colony motion (Fig. S1 in the Supplementary Information). The sum of the MS of the total 31 frames was divided by the 31 results in MMS (μm/hour), which produced a representative MS for the p1 cells. Validation of the OF algorithm used in this study was completed in the previous study by showing manual tracking of individual cells^29^.

### Correlation of the proliferative capacity of p1 oral keratinocytes with MMS using a standard protocol

**Figure 2:**
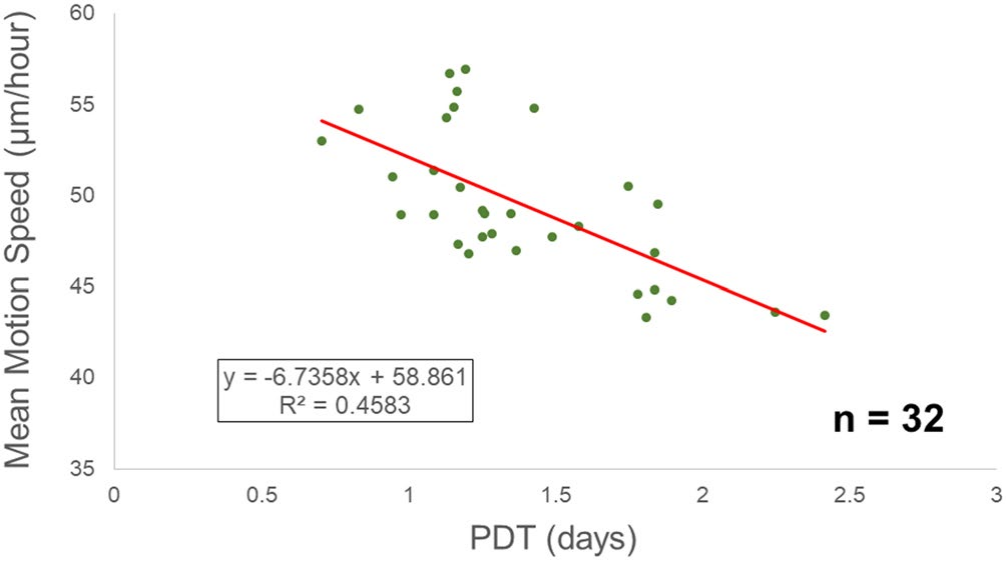
Correlation of the motion index of p1 oral keratinocyte cells/colonies with their proliferative capacity under standard cell culture protocol. Scatterplot showing the correlation between their MMS and proliferative capacity (PDT). The trend line is added, and its equation and the correlation coefficient are shown. (Spearman’s r =  − 0.6669, p < 0.0001, n = 32 individuals).

When the MMSs of all 32 samples were analysed and their PDTs were plotted on a scatterplot, the diagram showed a negative linear correlation with PDT, which was statistically significant (Fig. 2) (Spearman’s *r* = –0.6669, 95% confidence interval =  − 0.8274 to − 0.4057, *p* < 0.0001). Thus, oral keratinocyte p1 cells/colonies possessing a higher proliferative capacity were likely to have higher MMS values, thus indicating a higher locomotive ability, which is more robust correlation than the p0 cells/colony shown in our previous study^29^.

### Distinctive distribution of the MMS of p1 oral keratinocytes with their proliferative capacity under metabolic challenge protocols compared with that under the standard protocol

**Figure 3:**
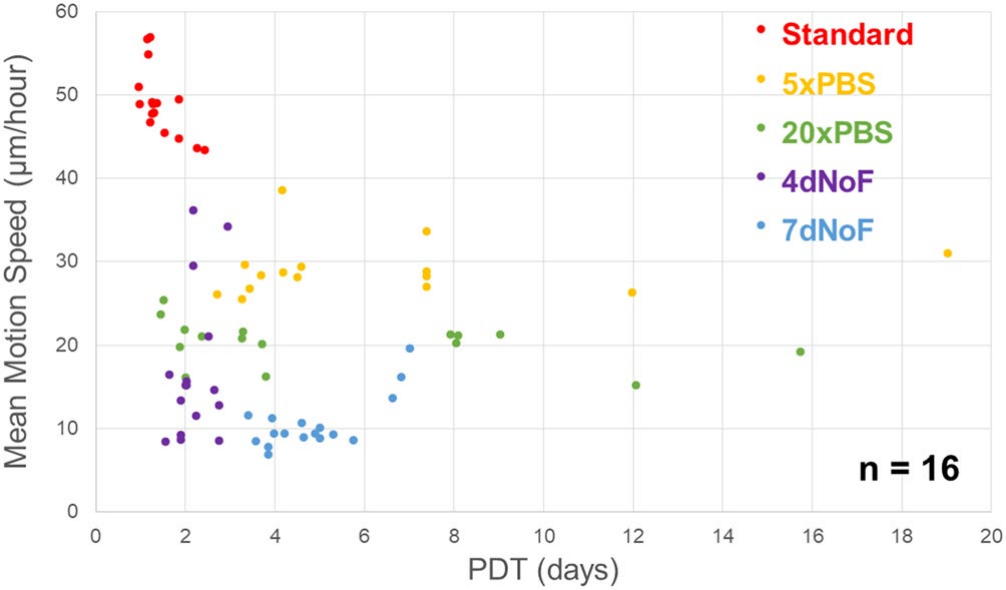
Distribution of the MMS of p1 oral keratinocytes with their proliferative capacity under metabolic challenge protocols compared with that under the standard protocol. Scatterplot showing the MMS and PDT of p1 oral keratinocytes cultured under two types of metabolic challenge protocols (low nutrition (5×PBS, 20×PBS) and no feeding (4dNoF, 7dNoF)), as well as the standard cell culture protocol. Distinctive pattern of sample distribution is present between cells cultured under standard protocol and metabolic challenges when grown in a 2D monolayer culture. (n = 16 individuals).

**Figure 4:**
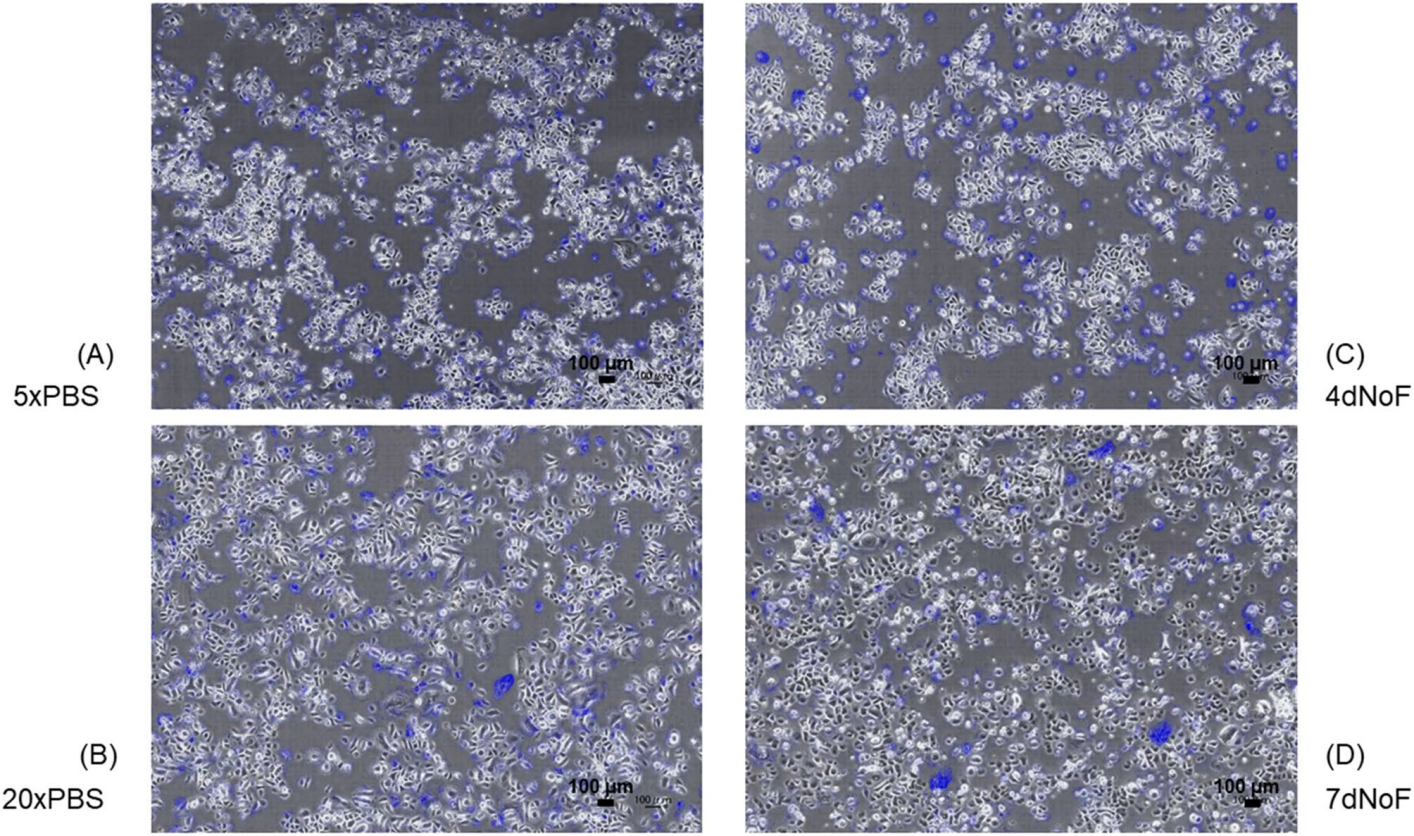
Cellular images having displacement vectors cultured under four different metabolic challenge protocols. Representative images (first frame) of cells in four different metabolic challenges with displacement vectors from MS calculation using OF algorithm. (**A**) 5×PBS, (**B**) 20×PBS, (**C**) 4dNoF, (**D**) 7dNoF. The original video files are provided in Supplementary Videos 1–6.

**Figure 5:**
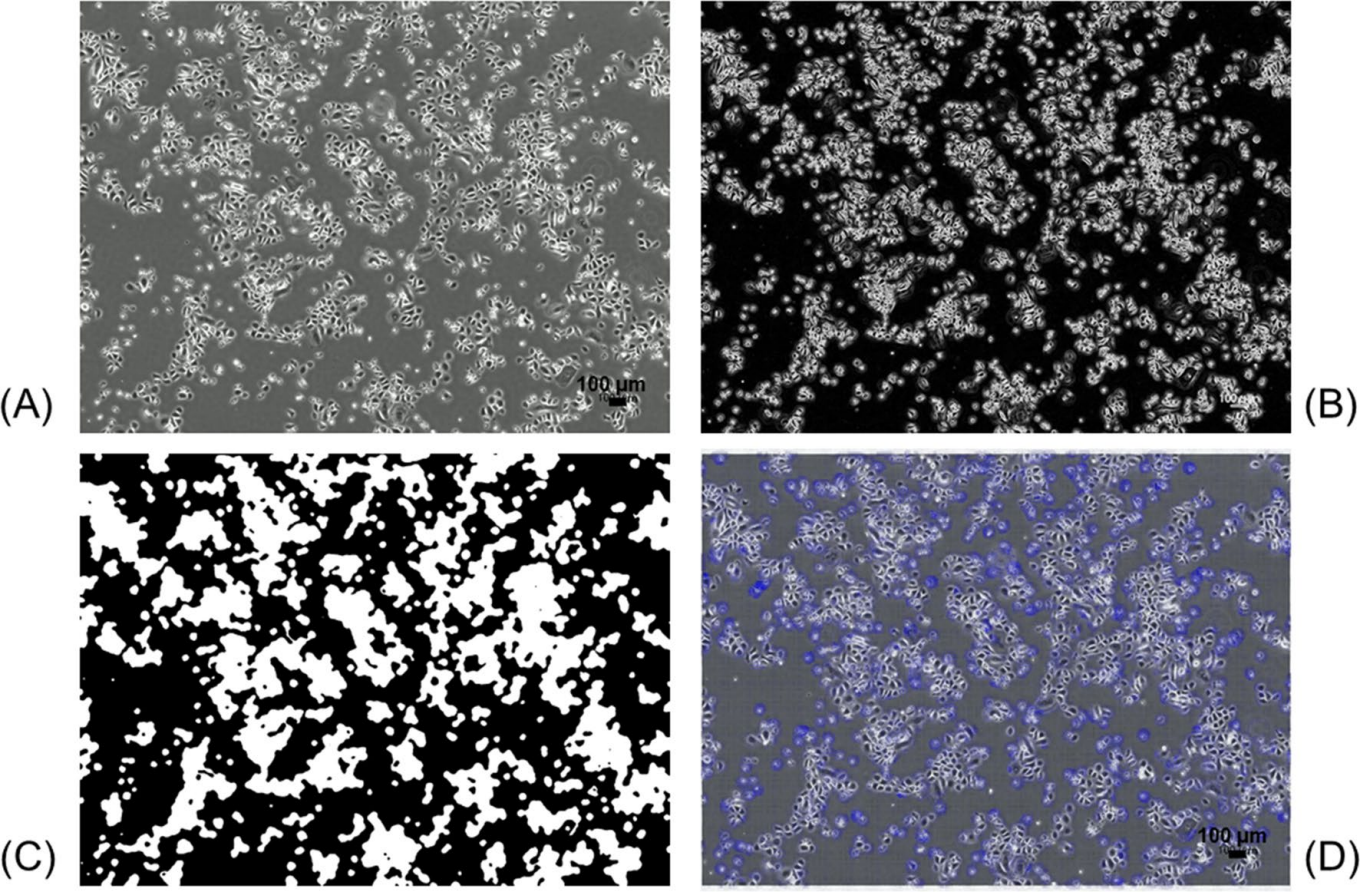
Images of pre-processing to calculate the MS by OF algorithm. (**A**) Representative original phase-contrast microscopic image of the p1 oral keratinocytes of the first frame of time-lapse microphotography. Cells are grown at approximately 50% confluence. (**B**) Illustration of edge detection for (**A**). Edges are detected by combining the horizontal and vertical derivatives using the square root of the sum of squares after 3 × 3 convolution kernels (Sobel filter) was applied to the image. (**C**) Illustration of the segmentation of cells/colonies by binarisation of (**B**). Black and white areas indicate background and cell area, respectively, by using the variational segmentation method of Chan and Vese. (**D**) Displacement vectors to be calculated by the OF algorithm in (**A**) are shown. By using the OpenCV library (ver.4.1), this full-screen image and the image at the next time step were used to calculate the MS as the mean value of the magnitude of vectors. The original video file is provided in Supplementary Video 6.

All of the MMS of p1 cells that received two types of metabolic challenges were plotted below the lowest MMS cultured under the standard protocol, which was 40 μm/h, thus demonstrating a distinctive distribution pattern of samples between cells cultured under standard and metabolic challenge protocols (Fig. 3). This differential difference was confirmed by the representative images and movies having vectors (Fig. 4A–D and Supplementary Video 1–4, compared with Fig. 5D and Supplementary Video 6). Because the p1 cells cultured under metabolic challenges were damaged during the 2D monolayer culture, the MMS of 40 μm/h is highly likely to be the specific level of MMS and to be used for differentiating the substandard p1 oral keratinocyte populations.

Under the low nutrition challenge, their MMS decreased in the order of 5×PBS and 20×PBS; however, the proliferative capacity of 5×PBS and 20×PBS, which were mostly lower than that of standard protocol, varied and overlapped (Figs. 3; 4A,B; Supplementary Video 1, 2). Under the no-feeding challenges, the majority of samples showed a similar MMS, which is much lower than the standard protocol (Figs. 3; 4C,D; Supplementary Video 3, 4). By contrast, although the proliferative capacity of 4dNoF was similar to that of the standard protocol, it was higher than that of 7dNoF.

### Histologic examination of the EVPOME received metabolic challenges compared with the standard protocol

**Figure 6:**
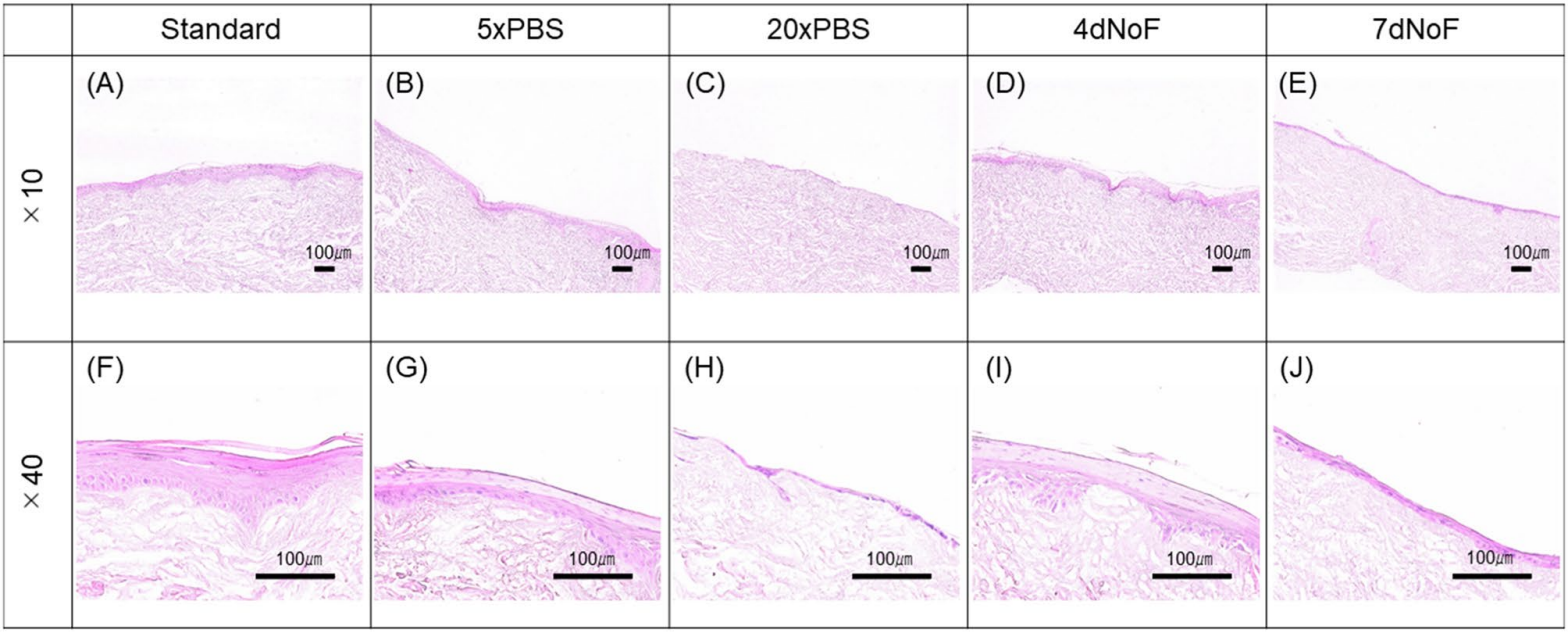
Histologic analysis of the EVPOME received metabolic challenges compared with the standard protocol. Representative histologic appearances of the EVPOMEs consisted of oral keratinocytes cultured under standard or four different metabolic challenge protocols in a 2D culture condition before transfer to a 3D culture condition. Oral keratinocytes grown in the standard protocol developed a well-differentiated, stratified epithelial layer on the scaffold (**A**,**F**). Compared with those histologic features, however, cells that received mild metabolic challenges, such as 5×PBS and 4dNoF, formed a thinner epithelial layer (**B**,**D**,**G**,**I**). Furthermore, cells that received severe metabolic challenges, such as 20×PBS and 7dNoF, generated a poorly differentiated epithelial layer (**C**,**E**,**H**,**J**). Additionally, some failed to generate a continuous epithelial layer. Haematoxylin and eosin staining. (n = 8 individuals) Original magnifications × 10 (**A**–**E**), × 40 (**F**–**J**). Scale bar = 100 μm.

We evaluated the histologic findings of EVPOMEs (n = 8) to further examine whether the MMS of the p1 cells/colony has the potential to predict an epithelial regenerative capacity and consequently to confirm whether the MMS of 40 μm/h is a detection threshold for monitoring the quality of the p1 cells before manufacturing an oral mucosa tissue-engineered construct, EVPOME. A well-differentiated, continuous stratified epithelial layer was developed when p1 cells cultured under the standard protocol (Fig. 6A,F) and cells under the 5×PBS and 4dNoF challenge protocols were seeded on AlloDerm (Fig. 6B,D,G,I). However, the epithelial layer of cells that received a challenge protocol was thinner and showed less distinct layers than that of the standard protocol, which suggests that their epithelial regenerative capacity was dampened (Fig. 6B,D,G,I). Moreover, although there was a continuous epithelial layer present in some of the EVPOMEs composed of cells cultured under 20×PBS and 7dNoF challenge protocols, some of the EVPOMEs showed more severe metabolic challenges, such as a discontinuous epithelial layer and complete failure to form the epithelium (Fig. 6C,E,H,J). Such poorly formed epithelial layers were not qualified for clinical use despite a continuous layer. The scatterplot of eight samples to examine the epithelial regenerative capacity showed a similar distribution pattern between cells cultured under standard and metabolic challenge protocols (Fig. S2 in the Supplementary Information).

### Proliferative activity of challenged oral keratinocytes in EVPOMEs

**Figure 7:**
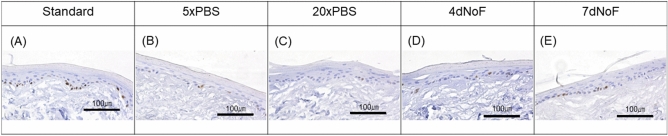
Ki-67 immunohistochemical analysis of the EVPOME received metabolic challenges compared with the standard protocol. Representative images of Ki-67 immunohistochemistry of the EVPOMEs. Ki-67-positive cells are mainly present in the basal layer. The number of Ki-67 positive cells was remarkably fewer when cells received metabolic challenges before development of the EVPOMEs. (**A**) Standard protocol, (**B**) metabolic challenge of 5×PBS, (**C**) metabolic challenge of 20×PBS, (**D**) metabolic challenge of 4dNoF, (**E**) metabolic challenge of 7dNoF. Original magnification × 40. Scale bar = 100 μm.

**Figure 8:**
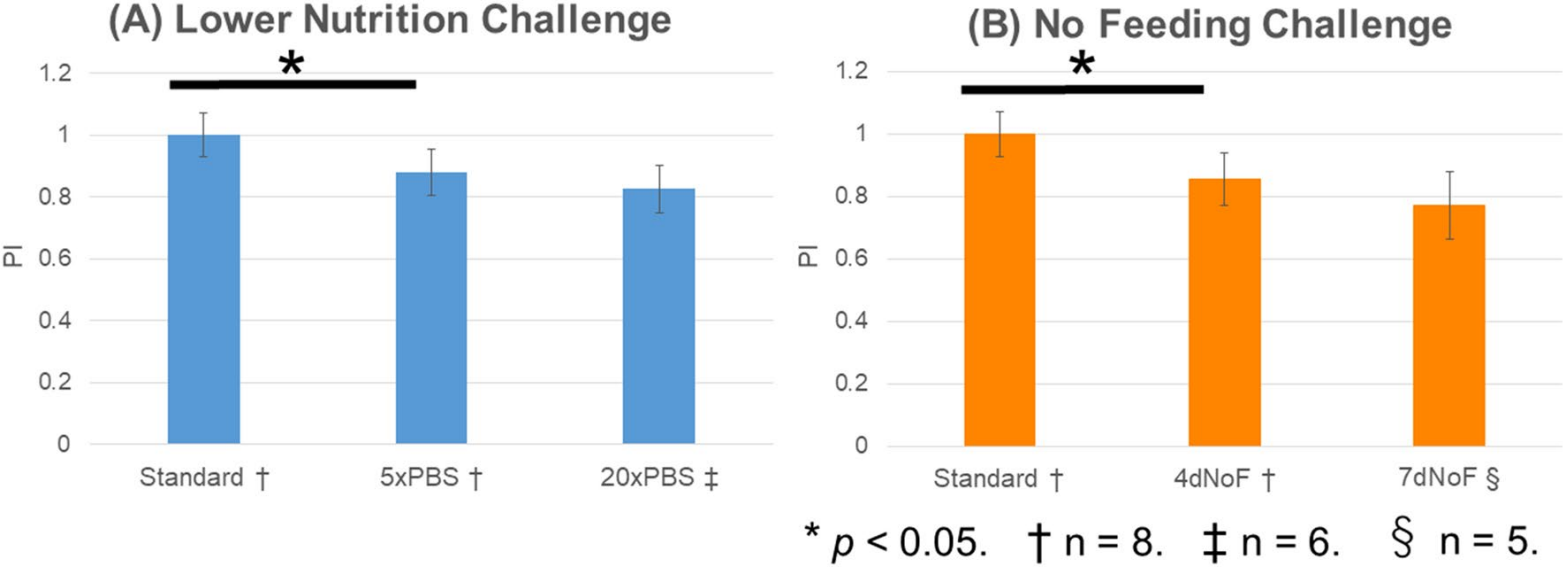
Proliferative index (PI) of EVPOMEs. (**A**) The PI of EVPOMEs between cells cultured under standard protocol and those that received the lower nutrition challenge. (**B**) The PI of EVPOMEs between cells cultured under standard protocol and those that received the no-feeding challenge. Data are presented as the mean ± standard deviation (SD). The statistical analysis was performed between the PI of the standard protocol and the PI of either the metabolic challenge protocol of 5×PBS or that of 4dNoF using a paired *t*-test. Because a continuous epithelial layer failed to be formed among some EVPOME specimens in which cells received severe metabolic challenges, such as 20×PBS and 7dNoF, the significant difference was obvious. * indicates statistical significance (*p* < 0.05).

Furthermore, to quantify the proliferative activity of EVPOMEs that had received metabolic challenges when cultured in a 2D monolayer, the proliferative index (PI) was calculated and compared with that of the standard protocol with Ki-67 immune-reaction (Fig. 7A–E). The result demonstrated that the PIs of EVPOMEs developed under 5×PBS and 4dNoF challenge protocols were significantly lower than that of the standard protocol (Fig. 8A,B). Additionally, the PIs of EVPOMEs developed under the 20×PBS and 7dNoF challenge protocols were much lower than the 5×PBS and 4dNoF of challenge protocols.

## Discussion

A cell/tissue-engineered product developed ex vivo for use in wound repair, such as EVPOME grafts and various medical therapies, require testing, monitoring and inspection to evaluate its quality from start to completion of the cell culture process, which finally goes through a release testing prior to transplantation into/onto the human body. Current popular technologies usually destroy part of the manufactured cells/tissues for monitoring and testing, although the cells/tissues assessed should be put into the body later on. Thus, non-invasive tools to assess the in vitro viability, metabolic activity and structures in real time are necessary to develop in regenerative medicine. Recent advances in optics and imaging analysis, such as time-lapse microscopic images combined with computational machine learning^4,37,38^ and the use of a specific sensor^39^, have provided many opportunities to address these issues for the QC of cells to be engineered. Previously, we applied two image analysis algorithms of OF and NCC to p0 oral keratinocytes during the early stage of 2D cell culture and confirmed that the two motion indices calculated by both algorithms were applicable and feasible for non-invasive monitoring of cells and can be used as an appropriate tool for QC^29^. This study aimed to improve our previous methodology and to expand its application for p1 cells and, hereafter, in a later stage culture as a non-invasive tool for QC. We found that our technique enabled us to evaluate the quality of p1 and subsequent passaged cells non-invasively in a total 2D cell culture period prior to manufacturing the 3D tissue-engineered construct, which allows for thorough monitoring.

The present study applied only the OF algorithm with binary image segmentation to the cell area of p1 cells/colonies for the full-screen analysis as a non-invasive and quantitative measurement, instead of using two different algorithms (OF and NCC). Due to the higher density and narrower spaces between the neighbouring cells/colonies, which are characteristic of p1 cell spatio-temporal growth kinetics compared with p0 cells, we implemented different segmentation imaging techniques that do not require extractions of the cell areas in which targeted colonies are present (conventional approach by using the threshold) before applying the OF algorithm. This pre-processing results in an absolute and immense increase in the background region. In contrast to the decrease in data accuracy of NCC when the microscopic image contains a large background region, the accuracy of OF analysis was not affected in such cases, because the OF algorithm is based on image brightness intensity tracking. Thus, the NCC algorithm was not used in this study in terms of the accuracy of the image analysis. As shown in Fig. 5D and Fig. S1, because displacement vectors to determine MS were successfully visualised and MS was satisfactorily calculated, OF algorithm is tolerable and applicable for quantitative and non-invasive measurement of the p1 cells/colony motion in this full-screen analysis.

Although the OF algorithm used in this study was the same as that used in the previous study, the image segmentation as pre-processing prior to applying OF algorithm to calculate MS were different to determine ‘cell area’. In this study, MS recognises the mean value of all the lengths of the displacement vector within the cell area, which were binarised, while it measures only the part where the displacement vector length is 1 pixel or greater than the cell area in the previous study. Technically, in the previous study, in case of the length of the displacement vector smaller than 1 pixel, the targeted area was recognised as the background, not the cell area, although cells moved a little. However, in this study, since the cell area to measure the overall cells/colony growth is directly and entirely visualised and detected by segmentation, MS is accurately analysed despite the image containing a mixture of moving and non-moving cells. Thus, the different approach for image segmentation used in this study is likely to increase the data accuracy of the measurement of cells/colony motion, thereby contributing to a higher correlation coefficient.

Due to the difference in the spatio-temporal proliferating pattern between p0 and p1 cells/colonies, in the present study, time-lapse microscopic imaging started when the cell confluency reached 50%, based on the outcome of overall cells/colony growth and changes in the MS over the 4 h period. Since the reduction of MS was notable when microphotography started at ≥ 70% confluency despite continuous cell proliferation over 4 h, it is highly likely to increase cell–cell contact, which would result in contact inhibition during measurement. By contrast, the MS slightly increased when the imaging started at 30% confluency. This suggested that the cell culture conditions, such as cell confluency at the start of microphotography, affect the MMS as the cells/colony motion index in terms of the definition of MMS in this study^40,41^. Thus, this protocol is appropriate and validated for use in the quantitative measurement of p1 cells/colony motion.

This study revealed that the MMS of p1 cells/colonies had a negative correlation with their PDT, which indicated that the faster those p1 cells/colonies move, the higher the proliferative capacity they have, which is similar to the findings of our previous study. Notably, their correlation was almost at a ‘strong’ level, which suggests higher accuracy of our outcomes under the protocols used in this study for a non-invasive tool for QC in oral keratinocytes regenerative medicine. In fact, when applying the current protocol to p0 cells/colonies, their MMSs were absolutely lower (data not shown). In the previous study, even though the same objective lens was used, we only analysed zoomed subparts of the image by scaling the limited area where target colonies were present. Thus, the current protocol created for the characteristic growth kinetics of p1 cells/colonies could enhance data accuracy, because the full-screen analysis renders the travelling distance of cells shorter. It also contributed to a decrease in the variation of MSs, since the cell motion ceases when cells divide.

This study was able to determine the threshold of MMS to efficiently differentiate substandard cell populations before manufacturing a tissue-engineered oral mucosa tissue construct by utilising two types of metabolic challenge protocols that are similar to protocols used in previous studies^18,19^. Obviously, as opposed to cells being cultured under the standard protocol, MMSs from cells that received metabolic challenges when grown in a 2D monolayer culture were plotted with an MMS lower than 40 μm/h, which depends on different culture conditions. Additionally, the MMSs of cells cultured under more severe metabolic challenge protocols, such as 20×PBS and 7dNoF, tended to be lower than those under milder metabolic challenges, although some overlapped. Accordingly, the distribution of PDT plots was mostly dispersed around 2 days of cell culturing under standard and metabolic challenge protocols, which indicates a correlation with their MMSs, although the PDTs overlapped among cells receiving two types of metabolic challenge protocols. Apart from the primary aim, the nutrition challenge affected MMS more distinctly than the no-feeding challenge did, whereas the no-feeding challenge affected PDT more remarkably. As a result, the MMS of p1 cells/colonies determined by the OF algorithm is reliable as a non-invasive and quantitative index for QC in regenerative medicine.

Most importantly, the histologic evaluation of this study was successful in demonstrating the significant potential of the MMS in predicting the epithelial regenerative capacity of p1 oral keratinocytes. As expected, in contrast to the development of a continuous and well-stratified epithelial layer consisting of cells cultured under the standard protocol, cells cultured under the metabolic challenge protocols generated a thinner epithelial layer in EVPOME or failed to form a continuous epithelial layer, which demonstrated a poor regenerative capacity. Moreover, the significantly lower PI index of EVPOME that consisted of cells receiving 5×PBS and 4dNoF in a 2D monolayer culture condition was in accordance with the histologic findings, which is similar to the correlation with MMS. Considered together, these assessments of the epithelial regenerative capacity will make the MMS of p1 cells/colonies more robust and predictable as a QC tool. These findings confirmed that MMS, which is a non-invasive tool for monitoring the status of the oral keratinocytes culture, can be an informative and quantitative index that ensures that p1 cells are suitable for EVPOME fabrication. Nonetheless, the histologic findings indicated that cells with an MMS lower than 40 μm/h under the metabolic challenges in case of our culture system did not completely lose epithelial regenerative capacity. Thus, an MMS threshold can be used to screen the quality of cultured cells, particulary 40 μm/h in this study; however, the specific value has to be determined by different culture conditions and laboratories.

However, a molecular mechanism for identifying the correlation between the locomotive ability and proliferative capacity of oral keratinocytes has not been elucidated. Although many factors/signalling pathways should be associated with those cellular characteristics, one that is associated with the epidermal growth factor (EGF) receptor/ligand system can control skin homeostasis and regulation of keratinocyte stem cells^42^. Since our culture medium contains EGF as a growth supplement, elucidation of the correlation between cells/colony motion and the proliferative capacity of the EGF receptor/ligand system may guarantee more advances in regenerative medicine of oral mucosa. In addition, it is essential to determine whether our protocol is applicable for predicting the regenerative potential of more passaged cells, other epithelial cells and different culture conditions, such as different culture medium and surface coating of culture vessels. In fact, the MMS of p2 and p3 oral keratinocytes showed a correlation with the epithelial regenerative capacity, similar to p1 cells (unpublished data). Moreover, because the epidermal keratinocyte stem cell colony was successufully identified by the analysis of spatial characteristics and velocity of cells, the regenerative potential appears to be predictable^43^. However, further research is necessary to make our technique versatile.

## Materials and methods

### Procurement of oral mucosa samples and culturing primary oral keratinocytes

The procurement of oral mucosa samples and procedure for oral keratinocyte cultures were described previously^29^. All methods were performed in accordance with relevant guidelines and regulations. The details were described in the Supplementary Methods (Supplementary Information).

### Time-lapse microscopic imaging

Under appropriate conditions, randomly-chosen five locations within the dish of the p1 cell culture were subjected to time-lapse observation. Phase-contrast images were taken at 8-min intervals for 4 h, until a total of 31 images were produced using a × 4 PlanFluor NA0.13 PhL objective lens. The images were converted to video files using a BZ-X analyser (Keyence) (Fig. 5A; Supplementary Video 5). More detailed information was provided in the Supplementary Methods.

### Determination of the cell area using image segmentation

Previously, we utilised a conventional segmentation method of applying the threshold to the intensity value by extracting areas containing targeted cells/colonies within the microscopic image. Briefly, using the MS as an index of cell locomotive ability, we selected areas with an MS of p0 cells/colonies that was equal to or greater than 1.00 pixel/frame, which corresponded to a p0 oral keratinocyte cells/colony area^29^. However, it is time consuming and laborious to apply to p1 cell cultures instead of p0 cells/colonies in the current experimental design. Hence, we implemented a different approach to achieve high-precision segmentation.

For image segmentation before determining the MMS of the p1 oral keratinocyte cells/colonies by using the OF algorithm, we used the variational segmentation method of Chan and Vese^44^ to identify the surface area covered by all cells/colonies. The algorithm developed by Chan and Vese for active contours is a flexible method that enables the segmentation of many types of images. It has been applied to segment biological images such as cells, and its details have been reported^44^. There are four parameters (λ1, λ2, ν and μ) in this algorithm, and the parameters should be determined by the user. Details of each parameter are explained in a previous study^45^. Tuning of parameters was conducted by comparing manual and automatic segmentations and was selected to minimise the deviation between manual and automatic segmentations as much as possible. The validation data used was the image data shown in Fig. 5A. In this study, the preferred settings are λ1 = 1.2, λ2 = 1.0, ν = 0.02 and μ = 0.8.

Cell segmentation is conducted using the following three steps:

Step 1. Smoothing: All frames are smoothed using a Gaussian kernel (size is 3 pixel × 3 pixel window).

Step 2. Detect edges: 3 × 3 convolution kernels (Sobel filter) are applied to the image and vertical and horizontal derivatives are generated. Edges are detected by combining the two derivatives using the square root of the sum of the squares (Fig. 5B).

Step 3. Segmentation of p1 cells/colonies: By applying the Chan–Vese algorithm to the obtained image, the image is binarised into the background and cell area (Fig. 5C).

In the Chan–Vese algorithm, the use of small-sized images is recommended, because computation is time consuming. However, since the image size obtained by current time-lapse microscopic imaging is high resolution (1920 pixel × 1440 pixel), the algorithm is programmed in Python language with CuPy^46^ to calculate based on the GPU (graphics processing unit). The runtime of Step 3 is approximately 10 min on a 1920 × 1440 resolution for cell images (31 frames) using a single GPU (GeForce GTX 1080 Ti, 11 GB) core on a common desktop PC (memory, 16 GB).

### Measurement of the overall cells/colony growth

Because of the implementation of a different image segmentation, it is necessary to determine what cell growth conditions of p1 oral keratinocytes are appropriate to start time-lapse imaging. The simplest way to evaluate cell growth kinetics is to measure the overall cells/colony growth^47^, which was obtained via micro-photographing cells grown in the same culture dish at four different cell confluencies of 30%, 50%, 70% and 90% using manual observation. From the binary image after segmentation, the overall cells/colonies growth (%) was calculated, which was obtained by dividing the surface area covered by all cells/colonies shown in the full-screen mode by the entire image size (in pixel) for each frame over 4 h.

### Determination of MMS of the p1 cells

The MS was determined in this study by using the identical OF algorithm of our previsou study^29^. A total of 31 sequence frames were created, in which vectors are drawn for each full-screen image (Fig. 5D; Supplementary Video 6). The following procedure was described in the Supplementary Methods.

### Sample size estimation

The minimum required sample size was a priori determined by power analysis using G*power software^48,49^. As a result, a total sample size of 26 was required by setting two-tails with an effect size of 0.5, a significance level of 0.05 and a power of 0.8^50^.

### Standard protocol for culturing p1 oral keratinocytes and evaluating the correlation between the motion index and proliferative capacity

To evaluate the correlation of the proliferative capacity with the MMS under the standard protocol according to the current experimental design, p1 cells grown at approximately 50% confluence in the 35-mm dish were subject to time-lapse microphotography. The mean MMS of the five locations was represented as the MMS of the sample (n = 32). Regardless of the confluency, 24 h after the completion of time-lapse microphotography, p1 cells were collected, and the number of cells was counted to determine the proliferative capacity. As a parameter of proliferative capacity, population doublings (PDs) and PDT of p1 cells were calculated as described in Supplementary Methods. Subsequently, a total 32 of MMS and PDT were plotted on the scatter plot to evaluate the correlation between MMS and PDT.

### Metabolic challenge protocols for oral keratinocyte culture

We hypothesised that there should be a threshold for the MMS of p1 cells that can be differentiated as substandard cell populations. We compared the proliferative capacity of p1 cells under standard protocol and under two types of metabolic challenge protocols that include a lower nutrition challenge created by the dilution of completed EpiLife with D-PBS (Wako Chemical, Osaka, Japan) by 1:5 (5×PBS) and 1:20 (20×PBS) and a no-feeding challenge without a fresh culture medium change for four days (4dNoF) and seven days (7dNoF). Different from the standard protocol, which was used as a control for the metabolic challenges, a density of 1.25 × 10^5^ cells was plated into a 35-mm dish for those challenges except for 20 × PBS, for which the cells were plated at a density of 1.5 × 10^5^ cells in a 60-mm dish with complete EpiLife medium. To secure random sampling, cells were alternately subject to culturing under metabolic challenge protocols using a total of 32 oral keratinocytes (n = 16).

To test a lower nutrition challenge protocol, the cells were fed every 2 days with complete EpiLife, and at approximately 50% confluency, they were fed with each medium diluted with D-PBS by the indicated ratio, respectively. The cells of 5×PBS in the 35-mm dish were fed every 2 days with the diluted medium and time-lapse micro-photographed 3 days later. By contrast, the cells of 20×PBS in the 60-mm dish were photographed under the time-lapse microscope 4 h after switching the diluted medium.

For the no-feeding challenge protocol, no medium change was made until they were subjected to time-lapse microphotography, which took three days for 4dNoF and six days for 7dNoF after the medium change, respectively.

Regardless of the confluency rate, 24 h after time-lapse microphotography, the p1 challenged cells were collected using the previously described method, and the number of cells was counted. PD and PDT were calculated using the formula provided in Supplementary Methods in which N = the number of challenged p1 cells collected, N_0_ = the cell number inoculated, which is 1.25 × 10^5^ for 5×PBS, 4dNoF and 7dNoF or 1.5 × 10^5^ for 20×PBS) and I = days in the culture of challenged p1 cells. A step-by-step chart of the four metabolic challenge protocols is shown in Supplementary Figs. S3A and 3B in the Supplementary Information.

### Manufacturing of ex vivo produced oral mucosa equivalents (EVPOME)

#### Histologic and immunohistochemical examination of EVPOMEs

##### Evaluation of the proliferative activity of challenged oral keratinocytes in EVPOME

Manufacturing of EVPOMEs, their histologic and immunohistochemical examinations and evaluation of the proliferative activity of challenged oral keratinocytes in EVPOME were described in the Supplementary Methods.

### Statistical analysis

To examine the strength of a linear association between MMS and PDT, Spearman’s correlation coefficient was calculated for non-normal distribution of the PDT, and the coefficient, *r*, and *p* values were determined using Prism 7.05 (GraphPad Software, San Diego, CA, USA). The results of PI are presented as the mean ± standard deviation (SD). The comparisons between the cells under the standard protocol and metabolic challenge protocols (5×PBS and 4dNoF) were examined using a paired *t*-test. A *p*-value < 0.05 was considered statistically significant.

### Ethical approval and patient consent

The use of human oral mucosa keratinocytes and the procurement procedure was approved by the Internal Review Board of the Niigata University Medical & Dental Sciences Hospital. Number: 2015–5018, titled ‘Translational research towards advanced regenerative medicine of oral mucosa: From bench to bed side’. Informed consent was obtained from all patients.

## Conclusions

In this study, we improved our imaging analysis method to monitor and evaluate the proliferative capacity of oral keratinocytes during the later phase of cell culture, specifically for p1 oral keratinocytes. The present study was successful in measuring p1 cells/colony motion by applying the OF algorithm non-invasively and quantitatively and demonstrated the correlation between the index of cell motion, MMS and the index of proliferative capacity, PDT. This was achieved by a different protocol to determine the cell area of p1 cells/colonies using image segmentation. This includes time-lapse microphotography starting at approximately 50% cell confluency and the fewer frames required for the full-screen analysis before applying the OF algorithm. Combined with the results of the MMS of cells receiving metabolic challenges during the 2D monolayer culture condition, we were able to determine an MMS threshold of approximately 40 μm/hour, being exclusively valid to our oral keratinocyte culture system, to differentiate the substandard p1 oral keratinocyte populations before manufacturing a tissue-engineered oral mucosa tissue construct. Additionally, our histologic findings of EVPOME confirmed that the MMS can be used to screen the quality of cells that have sufficient regenerative capacity. Considered together, even as a single parameter, MMS has the potential to predict future epithelial regeneration during a 3D culture and can be used as an informative tool and powerful supportive index for QC in oral keratinocyte regenerative medicine.

## Supplementary Information

Supplementary Information.

Supplementary Video 1.

Supplementary Video 2.

Supplementary Video 3.

Supplementary Video 4.

Supplementary Video 5.

Supplementary Video 6.

## Data Availability

The software and datasets generated and analysed during this study can be provided by the corresponding author upon reasonable request due to pending patent application.

## References

[1] Behaegel J, Ní Dhubhghaill S, Koppen C, Zakaria N (2017). Safety of cultivated limbal epithelial stem cell transplantation for human corneal regeneration. Stem Cells Int..

[2] Mosquera-Perez R (2019). Stem cells and oral surgery: A systematic review. J. Clin. Exp. Dent..

[3] Sasaki T (2019). Evaluation of cell viability and metabolic activity of a 3D cultured human epidermal model using a dynamic autoradiographic technique with a PET radiopharmaceutical. Sci. Rep..

[4] Sasaki K (2016). Non-invasive quality evaluation of confluent cells by image-based orientation heterogeneity analysis. J. Biosci. Bioeng..

[5] Nishimura K (2019). Live-cell imaging of subcellular structures for quantitative evaluation of pluripotent stem cells. Sci. Rep..

[6] Bajcsy P (2015). Survey statistics of automated segmentations applied to optical imaging of mammalian cells. BMC Bioinf..

[7] Kato R (2016). Parametric analysis of colony morphology of non-labelled live human pluripotent stem cells for cell quality control. Sci. Rep..

[8] Imai Y (2018). In-process evaluation of culture errors using morphology-based image analysis. Regener. Therapy.

[9] Smith D, Glen K, Thomas R (2016). Automated image analysis with the potential for process quality control applications in stem cell maintenance and differentiation. Biotechnol. Prog..

[10] Izumi K, Neiva RF, Feinberg SE (2013). Intraoral grafting of tissue-engineered human oral mucosa. Int. J. Oral Maxillofac. Implants.

[11] Izumi K, Feinberg SE, Iida A, Yoshizawa M (2003). Intraoral grafting of an ex vivo produced oral mucosa equivalent: A preliminary report. Int. J. Oral Maxillofac. Surg..

[12] Burillon C (2012). Cultured autologous oral mucosal epithelial cell sheet (CAOMECS) transplantation for the treatment of corneal limbal epithelial stem cell deficiency. Invest. Ophthalmol. Vis. Sci..

[13] Ohki T (2006). Treatment of oesophageal ulcerations using endoscopic transplantation of tissue-engineered autologous oral mucosal epithelial cell sheets in a canine model. Gut.

[14] Kim YH (2016). Comparative analysis of substrate-free cultured oral mucosal epithelial cell sheets from cells of subjects with and without Stevens-Johnson syndrome for use in ocular surface reconstruction. PLoS ONE.

[15] Barbagli G, Heidenreich A, Zugor V, Karapanos L, Lazzeri M (2020). Urothelial or oral mucosa cells for tissue-engineered urethroplasty: A critical revision of the clinical outcome. Asian J. Urol..

[16] Teodori L (2017). Three-dimensional imaging technologies: A priority for the advancement of tissue engineering and a challenge for the imaging community. J. Biophotonics.

[17] Zhou X (2013). Noninvasive real-time monitoring by alamarBlue during in vitro culture of three-dimensional tissue-engineered bone constructs. Tissue Eng. Part C Methods.

[18] Chen LC (2019). Optical metric assessed engineered tissues over a range of viability states. Tissue Eng. Part C Methods.

[19] Chen LC (2014). The potential of label-free nonlinear optical molecular microscopy to non-invasively characterize the viability of engineered human tissue constructs. Biomaterials.

[20] Jan ES (2012). Programming computer vision with python. Progr. Comput. Vis. Python..

[21] Huang Y, Hao L, Li H, Liu Z, Wang P (2017). Quantitative analysis of intracellular motility based on optical flow model. J. Healthcare Eng..

[22] Zahedi A (2018). Deep analysis of mitochondria and cell health using machine learning. Sci. Rep..

[23] Parrillla E (2013). Ciliary motility activity measurement using a dense optical flow algorithm. Conf. Proc. IEEE Eng. Med. Biol. Soc..

[24] Tamada A, Igarashi M (2017). Revealing chiral cell motility by 3D Riesz transform-differential interference contrast microscopy and computational kinematic analysis. Nat. Commun..

[25] Wong AOT (2020). Combinatorial treatment of human cardiac engineered tissues with biomimetic cues induces functional maturation as revealed by optical mapping of action potentials and calcium transients. Front. Physiol..

[26] Lee EK, Kurokawa YK, Tu R, George SC, Khine M (2015). Machine learning plus optical flow: A simple and sensitive method to detect cardioactive drugs. Sci. Rep..

[27] Czirok A (2017). Optical-flow based non-invasive analysis of cardiomyocyte contractility. Sci. Rep..

[28] Kinoshita K (2019). Automated collective motion analysis validates human keratinocyte stem cell cultures. Sci. Rep..

[29] Hoshikawa E (2019). Noninvasive measurement of cell/colony motion using image analysis methods to evaluate the proliferative capacity of oral keratinocytes as a tool for quality control in regenerative medicine. J. Tissue Eng..

[30] Lamb R, Ambler CA (2013). Keratinocytes propagated in serum-free, feeder-free culture conditions fail to form stratified epidermis in a reconstituted skin model. PLoS ONE.

[31] Oda D, Watson E (1990). Human oral epithelial cell culture I. Improved conditions for reproducible culture in serum-free medium. In Vitro Cell. Dev. Biol..

[32] Xu L, Schantz SP, Edelstein D, Sacks PG (1996). A simplified method for the routine culture of normal oral epithelial (NOE) cells from upper aerodigestive tract mucosa. Methods Cell Sci..

[33] Song J, Izumi K, Lanigan T, Feinberg SE (2004). Development and characterization of a canine oral mucosa equivalent in a serum-free environment. J. Biomed. Mater. Res. Part A.

[34] Morino T, Takagi R, Yamamoto K, Kojima H, Yamato M (2019). Explant culture of oral mucosal epithelial cells for fabricating transplantable epithelial cell sheet. Regener. Therapy.

[35] Izumi K, Takacs G, Terashi H, Feinberg SE (1999). Ex vivo development of a composite human oral mucosal equivalent. J. Oral Maxillofac. Surg..

[36] Izumi K, Terashi H, Marcelo CL, Feinberg SE (2000). Development and characterization of a tissue-engineered human oral mucosa equivalent produced in a serum-free culture system. J. Dent. Res..

[37] Matsuoka F (2013). Morphology-based prediction of osteogenic differentiation potential of human mesenchymal stem cells. PLoS ONE.

[38] Moen E (2019). Deep learning for cellular image analysis. Nat. Methods.

[39] Park K (2010). Measurement of adherent cell mass and growth. Proc. Natl. Acad. Sci. U.S.A..

[40] Garcia S (2015). Physics of active jamming during collective cellular motion in a monolayer. Proc. Natl. Acad. Sci. U.S.A..

[41] Tremel A (2009). Cell migration and proliferation during monolayer formation and wound healing. Chem. Eng. Sci..

[42] Nanba D, Toki F, Barrandon Y, Higashiyama S (2013). Recent advances in the epidermal growth factor receptor/ligand system biology on skin homeostasis and keratinocyte stem cell regulation. J. Dermatol. Sci..

[43] Hirose T, Kotoku J, Toki F, Nishimura EK, Nanba D (2021). Label-free quality control and identification of human keratinocyte stem cells by deep learning-based automated cell tracking. Stem Cells.

[44] Chan TF, Vese LA (2001). Active contours without edges. IEEE Trans. Image Process..

[45] Möller M, Burger M, Dieterich P, Schwab A (2014). A framework for automated cell tracking in phase contrast microscopic videos based on normal velocities. J. Vis. Commun. Image Represent..

[46] Okuta R, Unno Y, Nishino D, Hido S, Loomis C (2017). CuPy: A NumPy-compatible library for NVIDIA GPU calculations. Workshop ML Syst. NIPS.

[47] Scherf N (2012). Imaging, quantification and visualization of spatio-temporal patterning in mESC colonies under different culture conditions. Bioinformatics.

[48] Faul F, Erdfelder E, Lang AG, Buchner A (2007). G*Power 3: A flexible statistical power analysis program for the social, behavioral, and biomedical sciences. Behav. Res. Methods.

[49] Beck TW (2013). The importance of a priori sample size estimation in strength and conditioning research. J. Strength Condit. Res..

[50] Erdfelder E, Faul F, Buchner A, Lang AG (2009). Statistical power analyses using G*Power 3.1: Tests for correlation and regression analyses. Behav. Res. Methods.

