# Supplementary material for: Cells/colony motion of oral keratinocytes determined by non-invasive and quantitative measurement using optical flow predicts epithelial regenerative capacity
Source: Sci Rep. 2021 May 17;11:10403. doi: 10.1038/s41598-021-89073-y (PMC8128884; doi:10.1038/s41598-021-89073-y)
Supplement: Supplementary file 1 — Supplementary Information. [file 41598_2021_89073_MOESM1_ESM.docx]

**Cells/colony motion of oral keratinocytes determined by non-invasive and quantitative measurement using optical flow predicts epithelial regenerative capacity**

Emi Hoshikawa^1,2†^, Taisuke Sato^3†^, Kenta Haga^1^, Ayako Suzuki^1,4^, Ryota Kobayashi^1^, Koichi Tabeta^2^, Kenji Izumi^1*^

^1^ Division of Biomimetics, Faculty of Dentistry & Graduate School of Medical and Dental Sciences, Niigata University, Niigata, Japan

^2^ Division of Periodontology, Faculty of Dentistry & Graduate School of Medical and Dental Sciences, Niigata University, Niigata, Japan

^3^ Center for Transdisciplinary Research, Institute for Research Promotion, Niigata University, Niigata, Japan

^4^ Division of Pediatric Dentistry, Faculty of Dentistry & Graduate School of Medical and Dental Sciences, Niigata University, Niigata, Japan

† These authors contributed equally to this work

* Corresponding author:

Kenji Izumi (0000-0002-6302-6828)

Telephone: +81-25-227-2850

Fax: +81-25-227-2854

Email: [izumik@dent.niigata-u.ac.jp](mailto:izumik@dent.niigata-u.ac.jp)

**Supplementary Information**

**SI Figure captions**

**
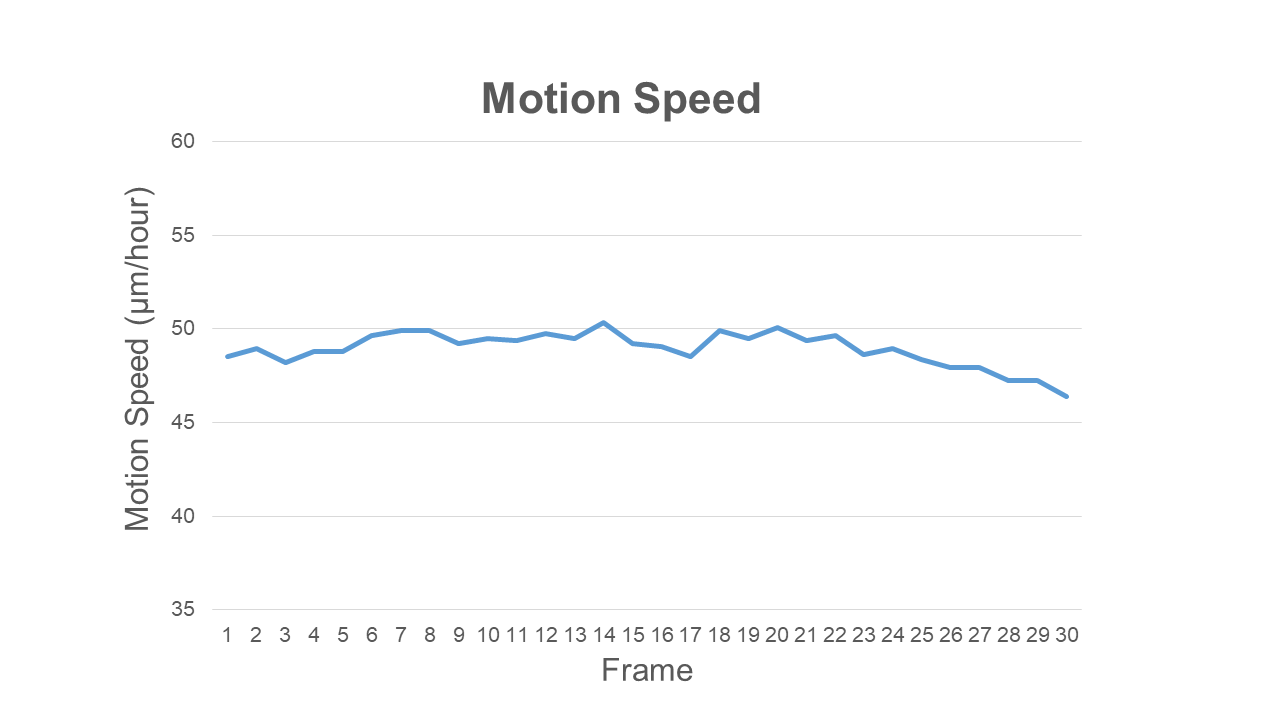
**

**Supplementary Figure 1. Motion Speed (MS) determined by the current time-lapse microphotography protocol using OF algorithm**

Representative change in MS of p1 oral keratinocytes to calculate MMS by 4-hour time-lapse microscopic observation (31 frames) when microphotography started at approximately 50% cell confluency.


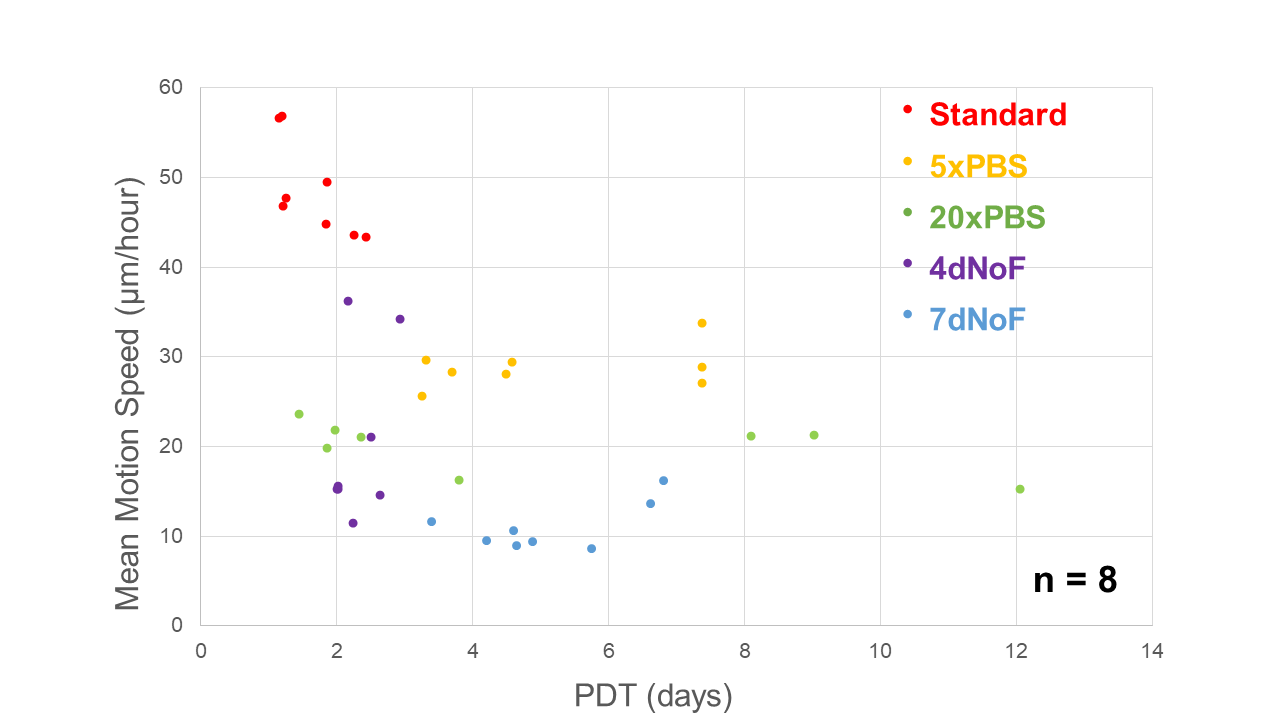


**Supplementary Figure 2. Distribution of the MMS of p1 oral keratinocytes with PDT under metabolic challenge protocols and the standard protocol used to develop EVPOMEs**

Scatterplot showing the MMS and PDT of p1 oral keratinocytes cultured under two types of metabolic challenge protocols (low nutrition and no feeding) and the standard cell culture protocol for the development of EVPOMEs to evaluate the epithelial regenerative capacity after receiving metabolic challenges (n = 8 individuals).

Distinctive pattern of sample distribution is similar to that in Fig. 3 (n = 16 individuals).

**
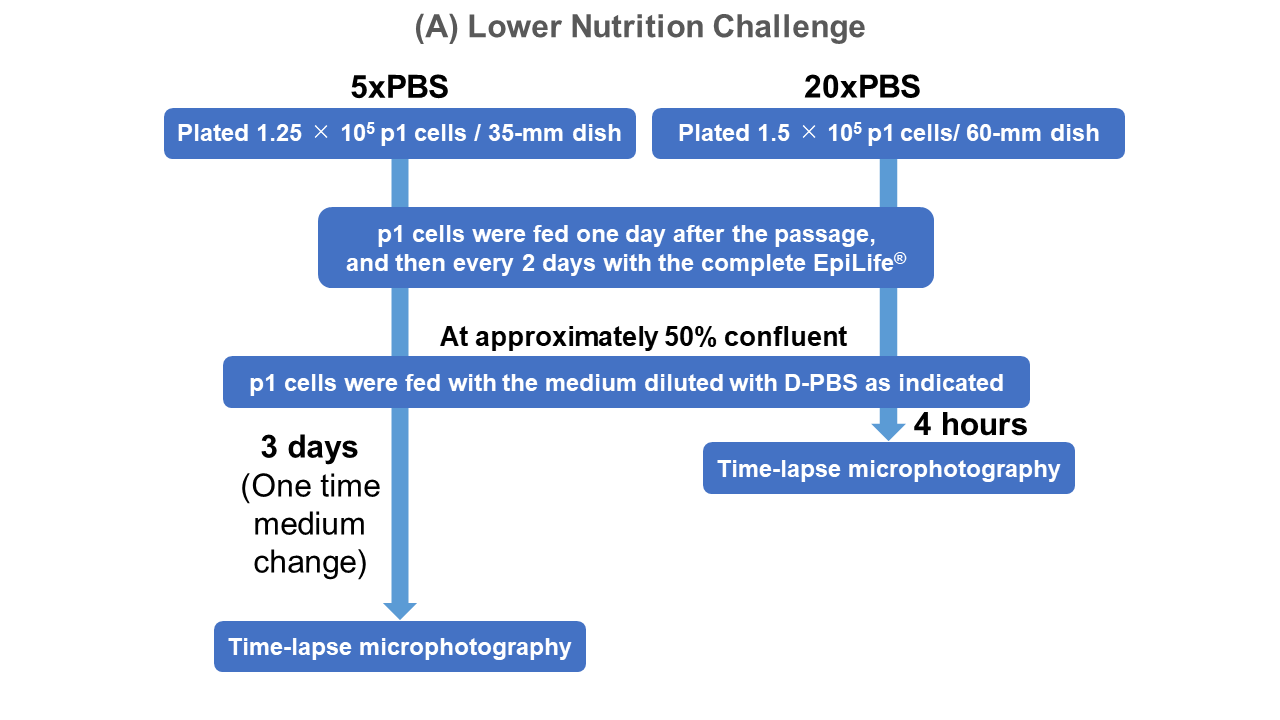
**

**
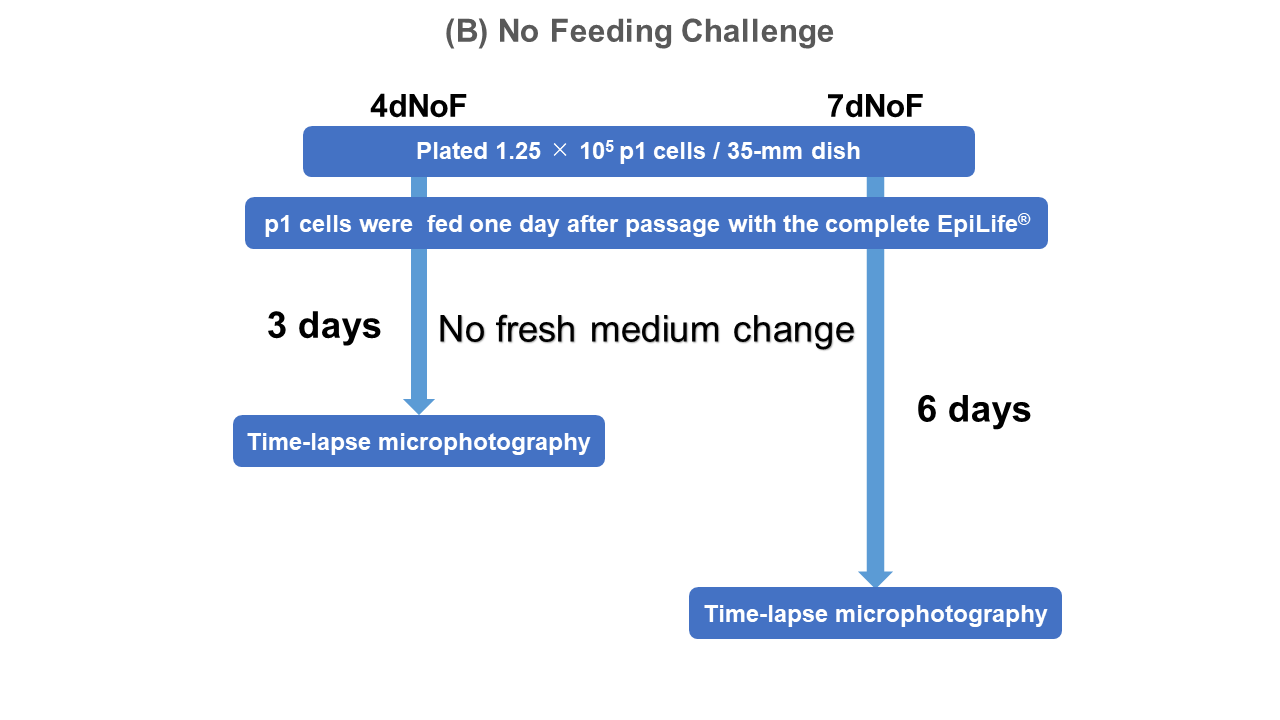
**

**Supplementary Figure 3. Step-by-step chart of metabolic challenge protocols for p1 oral keratinocytes**

**(A)** Low nutrition challenge protocol.

**(B)** No-feeding challenge protocol.

**Supplementary Video captions**

Representative video files with displacement vectors for MS calculation using OF algorithm, which are converted from phase-contrast images of cultured oral keratinocytes taken at 8 min intervals for 4 h (total 31 frames).

Supplementary Video 1: Cells were cultured under the metabolic challenge protocol of 5xPBS,

Supplementary Video 2: Cells were cultured under the metabolic challenge protocol of 20xPBS.

Supplementary Video 3: Cells were cultured under the metabolic challenge protocols of 4dNoF. Supplementary Video 4: Cells were cultured under the metabolic challenge protocols of 7dNoF.

The following videos are converted from phase-contrast images of cultured oral keratinocytes taken at 8 min intervals for 4 h (total 31 frames).

Supplementary Video 5: Representative video file before applying OF algorithm.

Supplementary Video 6: Representative video file with displacement vectors for MS calculation using OF algorithm.

**Supplementary Methods**

***Procurement of oral mucosa samples and culturing primary oral keratinocytes***

The procurement of oral mucosa samples and procedure for oral keratinocyte cultures were described previously.^29^ Demographic data of the 32 tissue samples include nine males with a mean age of 31.9 ± 12.4 years ranging from 20 to 61 years and 23 females with a mean age of 25.9 ± 7.9 years ranging from 17 to 49 years. The protocol for obtaining human oral mucosa tissue samples was approved by the Niigata University Hospital Internal Review Board (2015-5018). All methods were carried out in accordance with relevant guidelines and regulations. Briefly, mucosal tissue was digested overnight in a 0.025% trypsin/ethylenediaminetetraacetic acid (EDTA) solution (Thermo Fisher Scientific, Waltham, MA, USA). Oral keratinocytes were mechanically dissociated in the defined trypsin inhibitor (DTI; Thermo Fisher Scientific) and then seeded as p0 cells in ‘complete’ EpiLife^®^-containing EpiLife Defined Growth Supplements (Thermo Fisher Scientific), 0.06 mM Ca^2+^, gentamicin (5.0 μg/mL; Thermo Fisher Scientific) and amphotericin B (0.375 μg/mL; Thermo Fisher Scientific) at a density of 4.0–5.0 × 10^4^ cells/cm^2^. After reaching a confluence of approximately 80%, the p0 cells were detached with 0.025% trypsin/EDTA (Thermo Fisher Scientific), neutralised with DTI and plated as p1 cells at a density of 7.5 × 10^4^ cells in a 35-mm dish (Eppendorf) with complete EpiLife^®^ medium. On the following day, the p1 cells were fed with the complete EpiLife^®^ medium.

***Time-lapse microscopic imaging***

Under appropriate conditions, the dishes of the p1 cell culture were subjected to time-lapse observation. Cells were imaged with a Keyence BZ-X700 all-in-one fluorescence microscope equipped with a 5% CO_2_ temperature-coned chamber and time-lapse tracking system (Keyence, Osaka, Japan). We randomly chose five locations for each culture dish. Phase-contrast images were taken at 8-min intervals for 4 h, until a total of 31 images were produced using a ×4 PlanFluor NA0.13 PhL objective lens. The images were converted to video files using a BZ-X analyser (Keyence) (Fig. 5A; Supplementary Video 5). The video files were analysed using our image analysis software, which was based on the OF.

***Determination of MMS of the p1 cells***

The MS was determined in this study by using the identical OF algorithm of our previous study.^29^ A total of 31 sequence frames were created, in which vectors are drawn for each full-screen image (Fig. 5D; Supplementary Video 6). Pixels that move more have larger displacement vectors. Next, the MS of the cell/colony at frame *t* was calculated as

$$\text{MS}_{\text{t}}\text{ }\text{=}\text{ }\frac{\text{1}}{\text{N}}\sum_{\text{r}\text{∈}\text{A}}^{\text{N}} \left\| \text{v}_{\text{t}}\text{(}\text{r}\text{)} \right\|,$$

where MS*_t_* is the motion speed (pixel/frame) at frame *t*, **v***_t_*(**r**) is the displacement vector calculated using OF, **r** is a position vector, *N* is the total number of pixels added and A is the cell area obtained cell segmentation. Finally, the MMS was calculated by averaging the MS in all frames.

To convert the number of pixels into the distance that the target cells have moved, we used Image J (National Institutes of Health, Bethesda, MD, USA, http://imagej.nih.gov/ij/). The 100 μm scale bar on the movie was determined to be 53.3333 pixels, which resulted in 1.875 μm/pixel in these images. Since the interval time of each time-lapse movie is 8 min, the resulting MS in μm/h was calculated by multiplying the coefficient of 14.06 by the mean speed (pixel/frame) at frame *t*, which is equal to 1.875 multiplied by 7.5.

***The formula of PDT calculation:***

PDs = [log (N/N_0_)] [log 2]^−1^, PDT = I/PD; where N = number of cells collected, N_0_ = 7.5 × 10^4^, and I = days of culturing p1 cells.^35^

***Manufacturing of an ex vivo produced oral mucosa equivalent (EVPOME)***

To further investigate and compare the regenerative capacity of p1 cells correlated with a cell/colony motion index, EVPOMEs were manufactured by seeding p1 oral keratinocytes receiving metabolic challenge onto AlloDerm^®^ (Allergan, Madison, NJ, USA) according to the method described in our previous studies.^10,36^ The 2.0 × 10^5^ cells cultured under either standard or four different metabolic challenge protocols collected for the cell count were seeded onto AlloDerm^®^. To secure random sampling, cells were alternately subject to seeding onto the scaffold (n = 8) among 16 cells cultured under the metabolic challenge protocols. Briefly, after pre-soaking the AlloDerm^®^ with a type IV collagen solution (5 μg/cm^2^) (Sigma-Aldrich, St. Louis, MO, USA) in a well of 48 microwell plate (Corning Inc., Corning, NY, USA) overnight at 4°C, cells were seeded onto AlloDerm^®^. The composites were cultured in the complete EpiLife^®^ medium supplemented with 1.2 mM Ca^++^ for four days in a submerged condition and then raised to an air–liquid interface with the same culture medium for another 7 days. No metabolic challenges were performed during EVPOME manufacturing.

***Histologic and immunohistochemical examination of EVPOMEs***

A total of 40 EVPOMEs (obtained from eight individuals) were fixed with 4% paraformaldehyde in 100 mM D-PBS and embedded in paraffin. The paraffin-embedded samples were deparaffinised, rehydrated, cut into 5-μm thick sections and stained with haematoxylin and eosin for histologic examination. The sections were also prepared for immunohistochemical examination of Ki-67. The sections were deparaffinised in xylene and rehydrated in ethanol. Endogenous peroxidase was blocked with 0.3% hydrogen peroxide in methanol for 30 min. Antigen retrieval was achieved by an autoclave using 10 mM citric sodium buffer (pH 6.0) at 121°C for 10 min. After incubation with 5% milk protein in tris-buffered saline (TBS) for 30 min, sections were incubated with anti-mouse Ki-67 antibody (M7240) (Dako, Carpinteria, CA, USA) at a concentration of 1:100 at 4°C overnight. After washing with TBS, the sections were reacted with EnVision FLEX Plus (Dako) at room temperature for 1 h. The immunoreactions were then visualised with 3,3ʹdiaminobenzidine (Dojindo Co. Ltd., Kumamoto, Japan), and the sections were counterstained with haematoxylin.

***Evaluation of the proliferative activity of challenged oral keratinocytes in EVPOME***

To quantify the proliferative activity of p1 oral keratinocytes in EVPOMEs that had received metabolic challenges when cultured in a 2D monolayer, the ratio of Ki-67 positive cell as a proliferative index (PI) was calculated as described previously with modifications.***^1^*** First, five fields of the EVPOME specimens were randomly selected. Under the microscope (Keyence, Osaka, Japan) using a 10× PlanFluor NA0.30 PhL objective lens, the total numbers of basal keratinocytes and Ki-67-immunopositive basal keratinocytes were counted. The Ki-67 positive ratio (PR) was calculated by dividing the number of Ki-67-immunopositive cells by the total number of basal layer cells on each specimen, and the mean of the five specimens was represented as the PR of each EVPOME. The PI of the p1 oral keratinocytes was calculated by dividing the PR of each of the four different metabolic challenge protocols by the PR of the standard protocol.

**Supplementary　Reference**

1. Tobita, T., Izumi, K. & Feinberg, S. E. Development of an in vitro model for radiation-induced effects on oral keratinocytes. *International Journal of Oral and Maxillofacial Surgery* **39**, 364–370; 10.1016/j.ijom.2009.12.020 (2010).
